# MicroRNA-16-5p Inhibits Osteoclastogenesis in Giant Cell Tumor of Bone

**DOI:** 10.1155/2017/3173547

**Published:** 2017-05-15

**Authors:** Shang Sang, Zhichang Zhang, Shu Qin, Changwei Li, Yang Dong

**Affiliations:** ^1^Department of Orthopaedic Surgery, Sixth People's Hospital, Shanghai Jiao Tong University, Shanghai, China; ^2^Shanghai Key Laboratory for Bone and Joint Diseases, Shanghai Institute of Orthopaedics and Traumatology, Shanghai Ruijin Hospital, Shanghai Jiao Tong University School of Medicine, Shanghai, China

## Abstract

Giant cell tumor (GCT) of bone is an aggressive skeletal tumor characterized by localized bone resorption. MicroRNA-16-5p (miR-16-5p) has been reported to be downregulated in lesions of patients with GCT, but little is known about its role in GCT. To explore the underlying function of miR-16-5p in GCT, we first detected its expression in patients with GCT. The results showed that osteoclast formation increased, whereas miR-16-5p expression considerably decreased with the severity of bone destruction. Furthermore, we found that miR-16-5p expression considerably decreased with the progression of receptor activator of nuclear factor-*κ*B ligand- (RANKL-) induced osteoclastogenesis. Functionally, miR-16-5p mimics significantly reduced RANKL-induced osteoclast formation. However, treatment with an inhibitor of miR-16-5p significantly promoted osteoclastogenesis. These findings reveal that miR-16-5p inhibits osteoclastogenesis and that it may represent a therapeutic target for giant cell tumor of bone.

## 1. Introduction

Giant cell tumor of bone (GCTB) is a locally aggressive, osteolytic tumor that causes significant bone destruction at the epiphysis of long bones [1]. Both benign and malignant GCTBs have been described [2]. Although rarely lethal, GCTBs exhibit local recurrence in 27% to 65% of patients following primary surgical treatments [3], and up to 6% of GCTBs develop pulmonary metastases [4, 5]. Histologically, giant cell tumor (GCT) is a heterogeneous tumor that consists of three major cell types: osteoclast-like multinucleated giant cells, spindle-like stromal cells, and monocytic round cells [6]. These cells have different roles in the promotion of osteolysis such as cytokine secretion, or cellular interaction [7]. Since the hallmarks of GCT are its aggressive lytic behavior and the osteoclast-like tumor giant cells that play a crucial role in the lytic process [8, 9], the inhibition of osteoclastogenesis may represent a therapeutic approach for giant cell tumor of bone.

Recently, noncoding microRNAs (miRNAs) have emerged as important regulatory elements in the development of tumors. These are small (~20 nt) noncoding, single-stranded RNA molecules that negatively regulate their target genes by inducing mRNA degradation, or through the inhibition of translation [10]. miRNAs function by partially or completely binding to the 3′-untranslated region (3′-UTR) of their target mRNAs, thereby triggering either the inhibition of translation, or the degradation of the mRNA [11]. Besides its use as a potential biomarker to detect gastric cancer [12], miR-16-5p has also been shown to be stably expressed in breast cancer [13]. In addition, we have previously reported that miR-16-5p is significantly downregulated in GCT [14], although little is known about its role in GCT and osteoclast formation.

Here, we report that miR-16-5p expression significantly decreases with the severity of bone destruction and uncovers a crucial role for miR-16-5p in promoting the process of osteoclastogenesis.

## 2. Results

### 2.1. miR-16-5p Expression Is Downregulated in GCT

**Figure 1:**
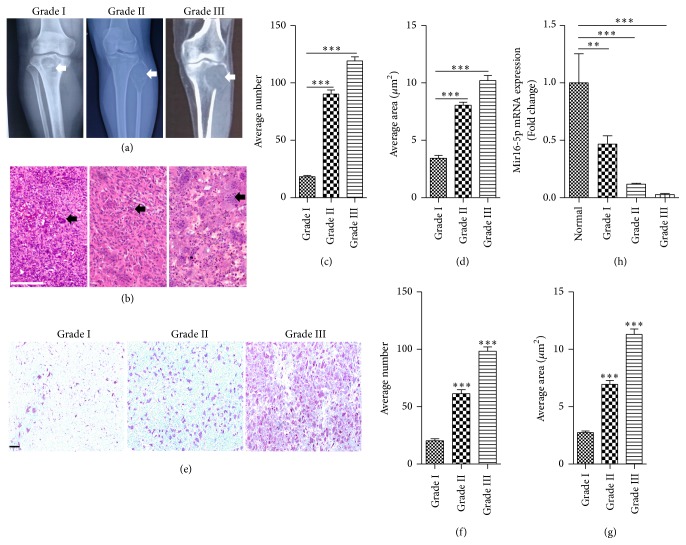
*miR-16-5p is downregulated in GCTB*. (a) Plain films of subjects in the GCT group showing different degrees of bone destruction (arrows indicated). (b) H&E staining of GCT slices in patients with different degrees of bone destruction. Panel (b) shows the multinucleated osteoclast-like giant cells (indicated by arrows) in the pathological images of patients for Campanacci Grades I, II, and III, respectively. Scale bars represent 10 *μ*m. (c) The average numbers of osteoclast-like multinucleated cells. (d) The average area of the osteoclast-like multinucleated cells. (e) Quantification of miR-16-5p mRNA expression in patients with GCT with different degrees of bone destruction. (f–h) TRAP staining of GCT slices in patients with different degrees of bone destruction. Scare bar represents 20 *μ*m. ^*∗∗∗*^*P* < 0.001  and  ^*∗∗*^*P* < 0.01.* P* values were analyzed using the one-way ANOVA test.

To investigate the underlying function of miR-16-5p in GCT, we first detected its expression in patients with GCTB. The results showed that osteoclast formation increased, whereas miR-16-5p expression decreased significantly with the severity of bone destruction (Figures 1(a)–1(h)). These results predicted that miR-16-5p might play a role in the pathogenesis of GCT.

### 2.2. miR-16-5p Inhibited the Process of Osteoclastogenesis

**Figure 2:**
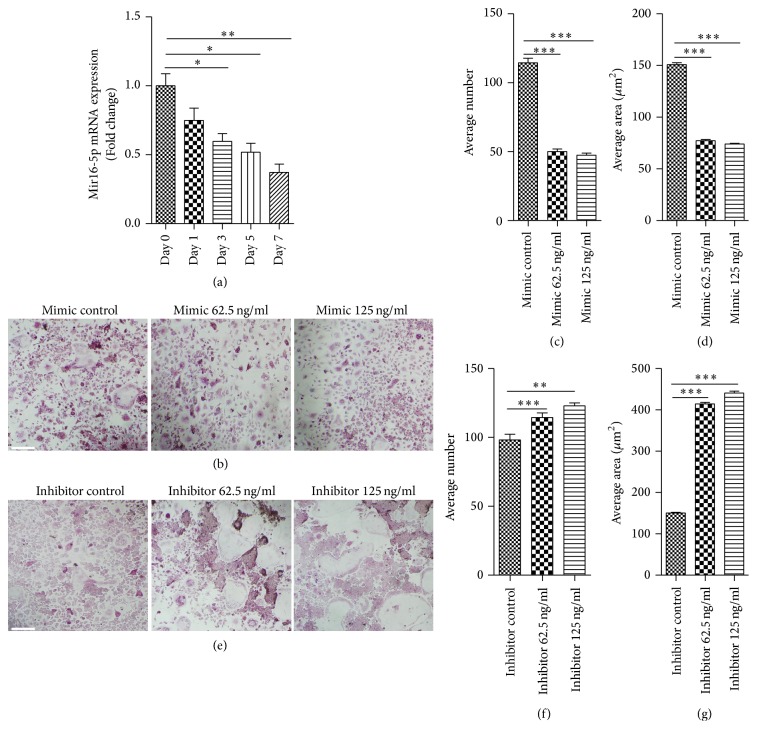
*miR-16-5p inhibits osteoclast formation*. (a) Quantification of miR-16-5p mRNA expression during RANKL-induced osteoclastogenesis. (b–d) TRAP staining showing a decrease in the number of osteoclasts following treatment with the miR-16-5p mimic. (e–g) TRAP staining showing an increase in the number of osteoclasts after treatment with the miR-16-5p inhibitor. Mimic represents the agomir for miR-16-5p, inhibitor represents the antagomir for miR-16-5p, mimic control represents the negative control for miR-16-5p mimic, and inhibitor control represents the negative control for miR-16-5p inhibitor. Scale bars represent 10 *μ*m. ^**∗**^*P* < 0.05, ^*∗∗*^*P* < 0.01, and  ^*∗∗∗*^*P* < 0.001.* P* values were analyzed using the one-way ANOVA test. All data are representative of three independent experiments.

**Figure 3:**
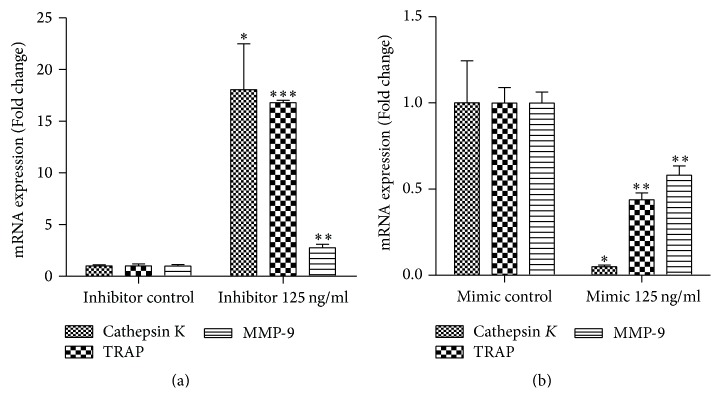
TRAP, CK, and MMP9* expression in RANKL-induced BMM osteoclastogenesis treated with miR-16-5p inhibitor or mimics*. (a) TRAP, CK, and MMP9 expression in RANKL-induced BMM osteoclastogenesis treated with miR-16-5p inhibitor. (b) TRAP, CK, and MMP9 expression in RANKL-induced BMM osteoclastogenesis treated with miR-16-5p mimics. Mimic represents the agomir for miR-16-5p, inhibitor represents the antagomir for miR-16-5p, mimic control represents the negative control for miR-16-5p mimic, and inhibitor control represents the negative control for miR-16-5p inhibitor. ^*∗*^*P* < 0.05, ^*∗∗*^*P* < 0.01, and ^*∗∗∗*^*P* < 0.001.* P* values were analyzed using the one-way ANOVA test.

**Figure 4:**
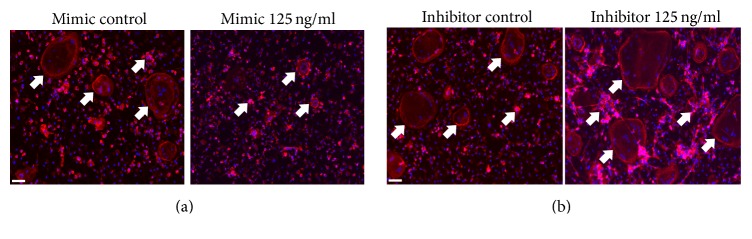
*Immunostaining of F-actin in RANKL-induced BMMs osteoclastogenesis treated with miR-16-5p inhibitor or mimics*. (a) Immunostaining of F-actin in RANKL-induced BMMs osteoclastogenesis treated with or without miR-16-5p mimics. (b) Immunostaining of F-actin in RANKL-induced BMMs osteoclastogenesis treated with or without miR-16-5p inhibitor. Arrows point towards the F-actin ring. Mimic represents the agomir for miR-16-5p, inhibitor represents the antagomir for miR-16-5p, mimic control represents the negative control for miR-16-5p mimic, and inhibitor control represents the negative control for miR-16-5p inhibitor. Scale bar represents 10 *μ*m. The original image magnification is 40x and the experiments were performed concomitantly.

Having observed that miR-16-5p expression was decreased in GCT, we next sought to explore the underlying function of miR-16-5p in the pathogenesis of GCT. Given that GCT is characterized by aggressive lytic behavior and by osteoclast-like giant tumor cells that play a crucial role in the lytic process, we hypothesized that miR-16-5p might play a vital role in the process of osteoclastogenesis in GCT. To test our hypothesis, we first measured miR-16-5p expression in RANKL-induced osteoclastogenesis of bone marrow-derived macrophages (BMMs). The results showed that miR-16-5p expression considerably decreased with the progression of RANKL-induced osteoclastogenesis, and the mRNA level of miR-16-5p decreased to 38% compared to the levels detected in the control group after seven days of RANKL stimulation (Figure 2(a)). Furthermore, a gain of function experiment revealed that a miR-16-5p mimic significantly inhibited RANKL-induced osteoclast formation (Figures 2(b)–2(d)), whereas a loss of function experiment revealed that the miR-16-5p inhibitor significantly enhanced RANKL-induced osteoclast formation (Figures 2(e)–2(g)). Consistent with the TRAP staining results, the expression of osteoclastogenesis-related genes, such as tartrate-resistant acidic phosphatase (TRAP), cathepsin K (CK), and matrix metallopeptidase 9 (MMP9), was detected by real-time PCR and further demonstrated that miR-16-5p functions as a suppressor of RANKL-induced osteoclast formation. The upregulated expression of TRAP, CK, and MMP9 induced by RANKL was significantly enhanced by a miR-16-5p inhibitor (Figure 3(a)), whereas these expression levels were substantially decreased when using a miR-16-5p mimic (Figure 3(b)). A well-polarized F-actin ring is required for mature osteoclast formation and efficient bone resorption [15]. Therefore, we performed F-actin ring staining to estimate the effect of miR-16-5p on osteoclastogenesis. The results showed that the miR-16-5p mimic disrupted the structure of the F-actin ring in a dose-dependent manner (Figure 4(a)), whereas the miR-16-5p inhibitor promoted the clear formation of the F-actin ring (Figure 4(b)). Taken together, these results showed that miR-16-5p inhibited the process of osteoclastogenesis in BMMs.

## 3. Discussion

GCTB is characterized by numerous osteoclast-like multinucleated giant cells that are primarily responsible for the extensive bone resorption by the tumor [16]. Although a number of studies have focused on the causes of GCT, the underlying pathology is not yet fully understood. Here we report that miR-16-5p was significantly downregulated in the lesions of patients with GCT. Furthermore, miR-16-5p levels were substantially decreased in vitro during the process of RANKL-induced osteoclastogenesis in BMMs. Moreover, our gain of function experiment demonstrated that a miR-16-5p mimic significantly reduced RANKL-induced osteoclast formation. However, treatment with an inhibitor of miR-16-5p clearly promoted osteoclastogenesis. Collectively, these findings reveal that miR-16-5p inhibits osteoclastogenesis and may function as a therapeutic target for giant cell tumor of bone.

As GCTs are known as bone destructive neoplasms, the inhibition of bone resorption has been confirmed as an effective therapeutic strategy to reduce the recurrence of GCT [17]. Recently, several effective preventative and nonsurgical interventions have been introduced. One large recent study indicates that bisphosphonate has been widely used in the clinical treatment of GCT, because of its protective effect against osteolysis [16, 18]. However, it has been reported that the long-term administration of bisphosphonates may lead to bone necrosis and atypical fractures in long bones [15]. Denosumab, a monoclonal antibody against RANKL, has recently been approved for use in bony metastasis, hypercalcemia of malignancy recalcitrant to bisphosphonates, and certain giant cell tumors. This drug is currently in phase II clinical trials, regarding its efficacy in osteolysis [19]. Denosumab is mostly effective due to its antiresorptive effects, as bone alkaline phosphatase did not decrease until a month after injection, while bone turnover markers immediately decreased [20]. Therefore, therapeutic avenues targeting receptor activator of nuclear factor-*κ*B (RANK)/RANKL and its downstream molecules might represent good options for the treatment of GCTB.

Osteoclastogenesis is an intricate multistep process that begins with the proliferation and commitment of mononucleated precursors and culminates in the formation of large bone-resorbing polykaryons [21]. Some osteolysis-related proteins that are produced during this process may be targeted for regulation by miRNAs [14]. It has been reported that miR-126-5p is significantly downregulated in spindle-like stromal cells of GCTs and affects osteoclast differentiation and bone resorption, by repressing the expression of matrix metalloproteinase 13 (MMP-13) [6]. In addition, Huang et al. reported that miR-30a can regulate the expression of RUNX2 by binding to its 3′-UTR, which regulates osteoclast differentiation and promotes osteolysis in GCTB [7]. A recent study also revealed that miRNA-106b inhibits osteoclastogenesis and osteolysis by targeting RANKL in GCTB [16]. Interestingly, even more miRNAs may modulate the production and function of the osteoclast. Our study revealed that miR-16-5p inhibits osteoclastogenesis in BMMs and provides additional proof that miRNAs may function as potential therapeutic targets for the treatment of GCTB. However, we are aware of the limitations in the experimental design of our study. First, although our results revealed that miR-16-5p functions as an inhibitor of osteoclastogenesis, the results were acquired in BMMs from C57 mice, and it is less well known whether miR-16-5p has the same function in human macrophages. In addition, the intratumor heterogeneity of GCTB still needs further illustration. Lastly, the mechanisms by which miR-16-5p regulates the process of osteoclastogenesis remain an important topic to be addressed by future studies.

In conclusion, our study provides evidence that miR-16-5p may play a crucial role in GCTB by inhibiting the process of osteoclastogenesis. Our findings implicate a potential use for miR-16-5p as a therapeutic target for the treatment of GCTB.

## 4. Materials and Methods

### 4.1. Clinical Samples

Radiological images and the clinical characteristics of 29 Chinese patients (22–78 years of age) with GCTB, as well as fresh specimens for 17 GCTB tumors were collected. Primary GCT tissues were isolated from tumor samples derived from tumor resections. The nontumor infected cancellous bones from the same patients with GCT were used as normal controls. The tissues were snap-frozen and stored in liquid nitrogen within two hours after surgical excision. All patients underwent resection for primary GCT in our hospital between 2012 and 2015. All patients with GCTB received extended curettage, with no adjuvant therapy. The clinical characteristics of all the patients with GCTB are summarized in Table 1. The progression of GCTB was evaluated using the Campanacci grading. The research was approved by the Ethics Committee of the Sixth People's Hospital of Shanghai and by that of the Jiao Tong University (Shanghai, China), and written informed consent was obtained from all participants.

### 4.2. miRNA Extraction

Total RNA was extracted from the GCT tissue (*n* = 17), the cancellous bone (*n* = 4), and the in vitro cultured cells using Trizol (Invitrogen, Carlsbad, CA, USA).

### 4.3. Cell Lines and Cell Culture

For primary cell cultures, BMMs were isolated from C57 mice. BMM cells were maintained in MEM (GIBCO) medium supplemented with 10% fetal bovine serum (HyClone) and grown in an incubator (37°C, 5% CO2).

### 4.4. qRT-PCR for mRNA and miRNA Analysis

qRT-PCR was performed using the iTaq™ Universal SYBR Green Supermix (Bio-Rad Laboratories, CA, USA) on a 7500HT Real-Time PCR System (Life Technologies, USA). Real-time PCR primers used for GAPDH are (forward: 5′-AGGTCGGTGTGAACGGATTTG-3′, reverse: 5′-TGTAGACCATGTAGTTGAGGTCA-3′); TRAP (forward: 5′-CACTCCCACCCTGAGATTTGT-3′, reverse: 5′-CATCGTCTGCACGGTTCTG-3′); CK (forward: 5′-GAAGAAGACTCACCAGAAGCAG-3′, reverse: 5′-TCCAGGTTATGGGCAGAGATT-3′); MMP-9 (forward: 5′-CTCAGAGATTCTCCGTGTCCTGTA-3′, reverse: 5′-GACTGCCAGGAAGACCTTGGTTA-3′).

### 4.5. Cell Transfection

The agomir (mimic), antagomir (inhibitor), and negative controls for miR-16-5p were purchased from RiboBio (RiboBio, Guangzhou, China). BMM cells were stimulated with RANK ligand (RANKL) and transfected with the agomir, antagomir, and the negative controls for miR-16-5p at different doses (0, 62.5, or 125 ng/ml). For transfection, the Lipofectamine® 3000 Reagent (Invitrogen, USA) was used according to the manufacturer's instructions.

### 4.6. TRAP Staining

TRAP staining and F-actin ring formation were used to assay osteoclastogenesis. For TRAP staining, cells were fixed and stained using the TRAP activity kit (Sigma, USA). TRAP-positive multinucleated cells containing three or more nuclei were counted as mature osteoclasts.

### 4.7. Cell Counting

We used ImageJ (National Institutes of Health, USA) to count the number and area of the target cells.

### 4.8. Statistical Analysis

All data are present as mean ± SEM. We did analyses of multiple groups by one-way ANOVA with Bonferroni posttest of GraphPad prism version 5. For all statistical tests, we considered* P* value < 0.05 to be statistically significant.

## Figures and Tables

**Table 1:** Characteristics of the 29 patients with GCT.

	Patients with GCTB
Age (years)	37.07 ± 13.27
Sex (male/female)	17/12
Disease history (months)	6.52 ± 3.55
Tumor size (cm)	5.24 ± 2.15
Tumor site (spine/limbs)	0/29
Primary/recurrent tumor	19/10
Resection (segment resection/curettage)	15/14
